# From the Thoracoscope to the Robot: The Evolving Landscape of Minimally Invasive Pulmonary Surgery

**DOI:** 10.3390/cancers18172785

**Published:** 2026-08-27

**Authors:** Raghav Chandra, Amanda Soe, Amartya Dave, Johannes R. Kratz

**Affiliations:** 1 Division of Thoracic Surgery, Department of Surgery, University of California San Francisco, San Francisco, CA 94143, USA; raghav.chandra@ucsf.edu; 2 School of Medicine, University of California San Francisco, San Francisco, CA 94143, USA; amanda.soe@ucsf.edu (A.S.); amartya.dave@ucsf.edu (A.D.)

**Keywords:** non-small-cell lung cancer, robotic video-assisted thoracoscopic surgery, minimally invasive surgery, radiomics, artificial intelligence, uniportal, single-anesthetic marking and resection

## Abstract

Surgical resection is the main curative option for non-small-cell lung cancer. Pulmonary resections have historically been performed through a highly morbid open thoracotomy. Minimally invasive approaches to pulmonary resection have revolutionized the field with reduced perioperative morbidity, including less postoperative pain, length-of-stay, and complications, while potentially enhancing oncologic outcomes. In its current generation, robotically performed video-assisted thoracoscopic surgery (R-VATS) offers numerous advantages over traditional VATS and is increasingly becoming the standard of care. In this review, we highlight the technical and clinical advantages of R-VATS compared to VATS and traditional thoracotomy. We reflect on emerging advancements in the utilization of the minimally invasive platform for single-anesthetic marking and resection of pulmonary nodules, the integration of artificial intelligence and radiomics to augment preoperative planning, and the expanding role of single-incision robotic surgery. Lastly, we discuss the financial considerations of implementing a robotic thoracic platform and identify opportunities to optimize costs.

## Simple Summary

Minimally invasive surgical approaches are now the mainstay in the treatment of resectable non-small-cell lung cancer. Robotic video-assisted thoracoscopic surgery (R-VATS) represents the future of pulmonary resection and is associated with numerous advantages over traditional VATS and open thoracotomy. In this review, we discuss the recent advancements in minimally invasive approaches to lung cancer resection, including single-anesthetic marking and resection, single-incision robotic techniques, and the implementation of artificial intelligence and radiomics to enhance surgical planning and safe resection. We also reflect on the financial and logistical costs of implementing a robotic thoracic platform and identify potential mitigation opportunities.

## 1. Introduction

Non-small-cell lung cancer (NSCLC) remains the leading cause of cancer-related mortality worldwide [1]. Surgical resection remains the main curative option and is the gold standard of treatment for early-stage disease. Lung cancer resections were, historically, highly morbid operations performed through an open thoracotomy. Minimally invasive video-assisted thoracoscopic surgery (VATS), which has been adopted since the 1990s, has revolutionized lung cancer surgery and is associated with significantly less postoperative pain, morbidity, and hospital length-of-stay [2,3]. Indeed, the National Comprehensive Cancer Network (NCCN) now recommends minimally invasive approaches for all patients without surgical contraindications [4]. In the modern era, robotic-assisted VATS (R-VATS) has gained substantial traction over traditional VATS and is often considered the new standard of care. Indeed, as of 2022, 59% of lobectomies in the United States are performed robotically [5].

R-VATS confers several advantages over traditional VATS, including superior visualization, better maneuverability with robotic arms compared to linear VATS instruments, and a lower risk of open conversion [6]. Furthermore, R-VATS may be associated with better oncologic surveillance, with greater nodal station sampling than VATS [7]. In conjunction with these technological advancements, the landmark findings of the CALGB140503 and JCOG0802 trials demonstrated that sublobar resections are non-inferior to lobectomies for early-stage, peripheral tumors [8,9]. Perioperative immunotherapy for locally advanced NSCLC has also provided an important new group of patients who were traditionally deemed unresectable, and are now potential surgical candidates [10]. Altogether, these evolutions in our understanding and treatment of lung cancer set the stage for the next generation of precision oncology efforts with faster diagnosis, superior characterization of pulmonary lesions, and less invasive curative-intent surgery.

This review synthesizes the recent developments in minimally invasive pulmonary surgery. We discuss how the use of the robotic platform and the renewed emphasis on early diagnosis and treatment of early-stage NSCLC synergistically promote precise and efficient treatment, including the advent of single-anesthetic management of solid pulmonary nodules, advanced imaging and marking modalities to facilitate safe sublobar resection, the emergence of uniportal approaches to further minimize operative morbidity, and the future of minimally invasive lung cancer surgery with respect to cost and widespread implementation.

## 2. The Rise of the Robot: Technical Considerations and Advantages of the da Vinci Robotic Platform for Pulmonary Resection

The da Vinci Surgical System (Intuitive Surgical, Sunnyvale, CA, USA) is the predominant robotic platform for thoracic surgery. This robotic platform has several technical features that make it ideally suited for complex pulmonary resection. Fundamentally, the robotic platform consists of three major components: the Surgeon Console, the Patient Cart, and the Vision Cart. The Surgeon Console is where the surgeon sits and controls the robotic instruments on the Patient Cart. The Patient Cart contains the robotic arms that are docked to thoracoscopic ports (typically three or four, alongside an AirSeal assist port (CONMED, Utica, NY, USA)). EndoWrist instruments are attached to these arms and can perform complex intracorporeal maneuvers with seven degrees of freedom, which is markedly superior to the linear stick-based instruments used in traditional VATS. In addition, EndoWrist instruments eliminate natural tremors of the human hand. The Vision Cart houses the monitors, three-dimensional endoscope, light source, and core computer of the robotic system. A unique advantage of this robotic system is the ability to connect a second Surgeon’s Console to the system, which allows for real-time, dynamic instruction and guidance to assistant surgeons and surgical trainees, a feature that is distinct from a traditional VATS approach.

The robotic platform offers several advantages over traditional thoracotomy or VATS for pulmonary resection. A recent landmark meta-analysis of 578 studies demonstrated that minimally invasive approaches are associated with improved survival compared to open surgery (HR 0.83, 95% CI: 0.78–0.89), providing Level 1 evidence of the clear advantages of minimally invasive approaches [11]. Even between different MIS approaches, R-VATS confers several advantages over the VATS platform. Inherently, R-VATS provides significantly superior visualization of the operative field with much higher resolution, 3-D reconstruction, and the ability of the surgeons to control the camera themselves throughout the operation.

Because of these advantages, R-VATS approaches may be less morbid than traditional VATS. The PORTaL multicenter study investigated 5721 cases comparing open, VATS, and R-VATS approaches for Stage IA-IIIA tumors and found that R-VATS was associated with shorter operative time compared to VATS. Furthermore, postoperative transfusion rate and open conversion rates were significantly lower in the R-VATS cohort, particularly in patients who had undergone neoadjuvant therapy [12,13]. As neoadjuvant therapy has evolved in both its composition (including targeted therapy and immunotherapy) and its frequency of use over the past several years, this latter point is of critical importance. We previously highlighted that post-neoadjuvant immunotherapy pneumonitis and fibrosis may significantly complicate minimally invasive pulmonary resection [10]. Indeed, it is the authors’ experience that post-neoadjuvant fibrosis and treatment effect are frequently associated with more time-consuming and difficult hilar and sub-hilar dissections. It is this particularly high-risk cohort for which R-VATS may be advantageous. A recent propensity-matched analysis by Li et al. comparing R-VATS and VATS after neoadjuvant therapy (including chemotherapy and chemoimmunotherapy) demonstrated superior nodal harvest with shorter operative time, blood loss, and fewer postoperative air leaks. Furthermore, R-VATS was found to be superior to conventional VATS for complex sleeve resections and was associated with reduced operative time and chest tube duration [13]. These findings are further corroborated by a large high-level meta-analysis of 11,247 patients which found, in addition to lower blood loss and open conversion rates, reduced postoperative chest tube duration [14]. No differences in mortality, DFS, or OS were noted between cohorts [14].

R-VATS may also facilitate more comprehensive lymph node assessment compared to VATS. The aforementioned meta-analysis by Ma et al. also demonstrated that the R-VATS approach was associated with a greater number of harvested lymph nodes [14]. The RVlob trial randomized 320 patients to R-VATS vs. VATS, and demonstrated, with Level 1 evidence, that patients who underwent robotic resection had higher median total lymph nodes harvested (11 vs. 10; *p* = 0.02) and total lymph node stations (six vs. five; *p* < 0.001). Importantly, patients undergoing R-VATS had significantly higher N1 (hilar) lymph node sampling (six vs. five; *p* = 0.005), which is of critical importance for accurate postsurgical staging [7]. It is important to note that comfort and experience with one approach or another at the level of an individual surgeon impacts the degree of nodal harvest [15]. However, many surgeons find the robotic platform, with four working arms, three-dimensional visualization, and seven degrees of freedom of the robotic instruments, superior to VATS in meticulously dissecting hilar and mediastinal lymph nodes.

## 3. Two for One: Implementation of a Single-Anesthetic Strategy for Marking and Minimally Invasive Resection of Pulmonary Nodules

A major advancement in the care of early-stage lung cancer is the advent of single-anesthetic localization and resection of pulmonary nodules. It is well-established that even short delays from lung cancer diagnosis to surgical resection are associated with inferior outcomes [16]. Furthermore, difficulty in identifying smaller, ground-glass, and deeper nodules by VATS may lead surgeons to perform unnecessarily extensive resections or even convert to open when a sublobar resection would have sufficed. Indeed, a landmark prior investigation demonstrated that the failure probability of successfully resecting a ≤10 mm nodule more than 5 mm from the pleural surface using VATS without preoperative marking was 63% [17]. As such, the minimally invasive hybrid robotic platform is ideally suited to facilitate this consolidated approach.

**Figure 1:**
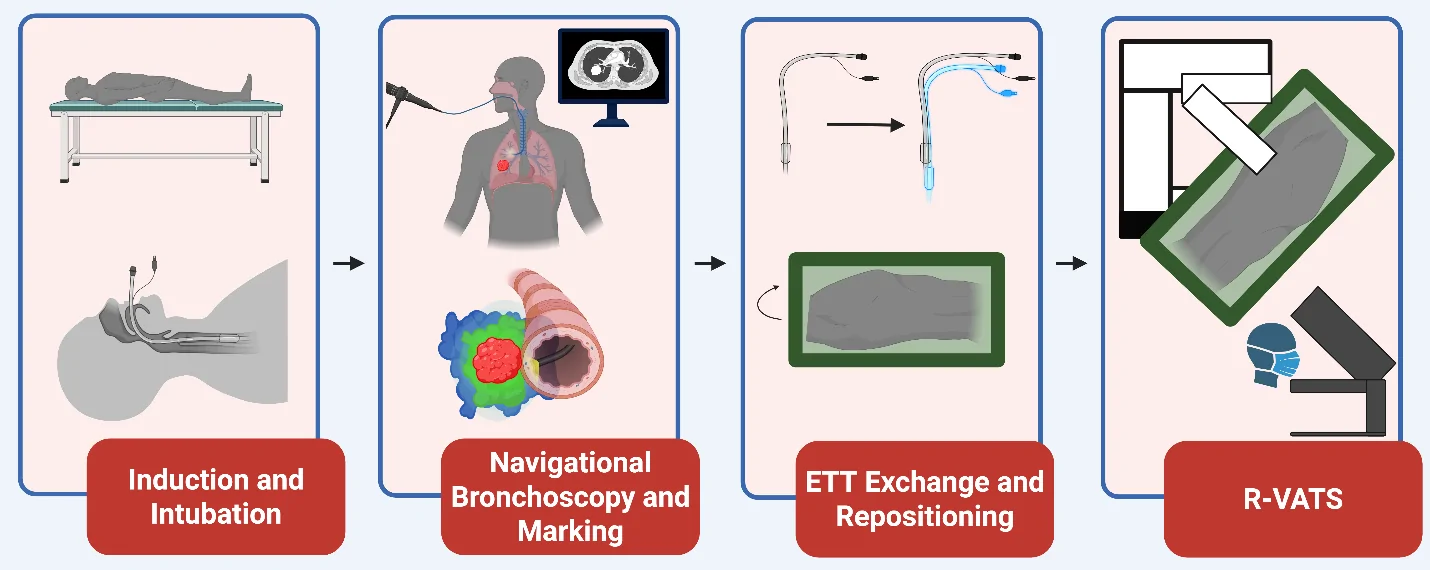
Workflow of single-anesthetic pulmonary nodule marking and resection. The patient is first positioned supine and intubated with a single-lumen ETT. Navigational bronchoscopy is then performed by the IP team, and the nodule is double-marked with indocyanine green and methylene blue. The patient is then re-intubated with a double-lumen ETT and repositioned in lateral decubitus. R-VATS is then performed by the cardiothoracic surgical team.

There are numerous approaches to marking a pulmonary nodule, including CT-guided percutaneous placement of radiotracer dyes or clips, endoscopic approaches such as fluoroscopic marking, electromagnetic navigational bronchoscopy (EMB), and robotic navigational marking. This novel approach has gained significant traction both in private practice and academic settings. At our institution, the interventional pulmonology (IP) and cardiothoracic surgical teams work closely to facilitate single-anesthetic resection with the following workflow: after induction with a single-lumen endotracheal tube (ETT), the IP team docks the robotic navigational platform or the EMB platform (determined case-by-case), localizes and marks the target nodule, and samples central lymph nodes as clinically indicated. Typically, both indocyanine green (ICG) and methylene blue are utilized for nodule marking. Once marking is complete, the bronchoscopy tower is withdrawn, and the patient is re-intubated with a double-lumen ETT. The patient is repositioned for surgery, and the R-VATS is carried out in standard fashion. Methylene blue is readily visible thoracoscopically, and the da Vinci surgical platform includes Firefly fluorescence visualization settings on the vision console, which facilitate seamless visualization of the ICG marking to augment localization of the nodule (Figure 1). Furthermore, during segmentectomies, intravenous ICG is administered to delineate perfused and nonperfused parenchyma after vascular division, facilitating parenchymal division. We typically send all sublobar resection specimens from these cases for frozen section to confirm capture of the target lesion.

The notion of single-stage localization and resection has been explored in the past, but its implementation in the pre-robotic era was highly variable. An initial proof-of-concept study demonstrated single-stage CT-guided localization with VATS resection in a hybrid suite in 12 patients with concerning solitary nodules, with a success rate of 10 out of 12 patients [18]. Utilizing fluoroscopic bronchoscopy, Yang et al. reviewed 85 nodules in 74 patients who underwent simultaneous nodule marking followed by VATS and found that the median duration of fluoroscopy was 3.3 min and the total median operating time was just under 3 h [19]. Importantly, the authors identified no significant intervention-associated complications [19]. Such investigations served as the platform for hybrid robotic marking and resection protocols.

In the robotic era, a recent pilot study of 12 patients demonstrated that the average turnover time from ION robotic navigational marking (Intuitive Inc., Sunnyvale, CA, USA) to R-VATS resection was 33 min, saving an average of 231 min per case [20]. This proof-of-concept study paved the way for a recent larger analysis by Ali et al. who studied outcomes after development of the “Robotic One Anesthetic Marking and Resection (ROMAR)” pathway. This study included 111 patients with 77.3% subsolid nodules (14.3% were ground-glass) that were marked with robotic bronchoscopy followed by sublobar robotic resection. The authors noted a success rate of 89.2% [21]. This study, the largest to date on this issue, definitively establishes ROMAR as a viable and effective technique for single-stage marking and minimally invasive resection of pulmonary nodules. While logistical and cost-related concerns will certainly impact widespread utilization of the combined approach, we anticipate that ROMAR or related hybrid single-stage techniques will become the standard of care for the timely treatment of patients with suspicious pulmonary nodules.

## 4. All for One: Uniportal VATS and the Rise of Single-Incision Approaches in R-VATS

It is well-established that VATS or R-VATS are substantially less morbid than open thoracotomy. Uniportal surgical approaches may reduce operative morbidity even further [22]. Uniportal minimally invasive thoracic surgery is not a novel concept and was first described more than 20 years ago [23,24,25]. This technique typically involves a 3–5 cm incision through which a single larger port is advanced into the pleural space, and linear instruments are coaxially passed alongside the thoracoscope. This approach is mechanistically distinct from traditional VATS due to essentially restoring the intrathoracic triangulation of instruments as would be done through an open approach. Uniportal VATS is associated with improved cosmesis, reduced postoperative pain [26], and equivalent postoperative complications and oncologic outcomes [26,27] compared to VATS. However, very steep learning curves and longer operative times have markedly precluded its widespread implementation [22,28].

**Figure 2:**
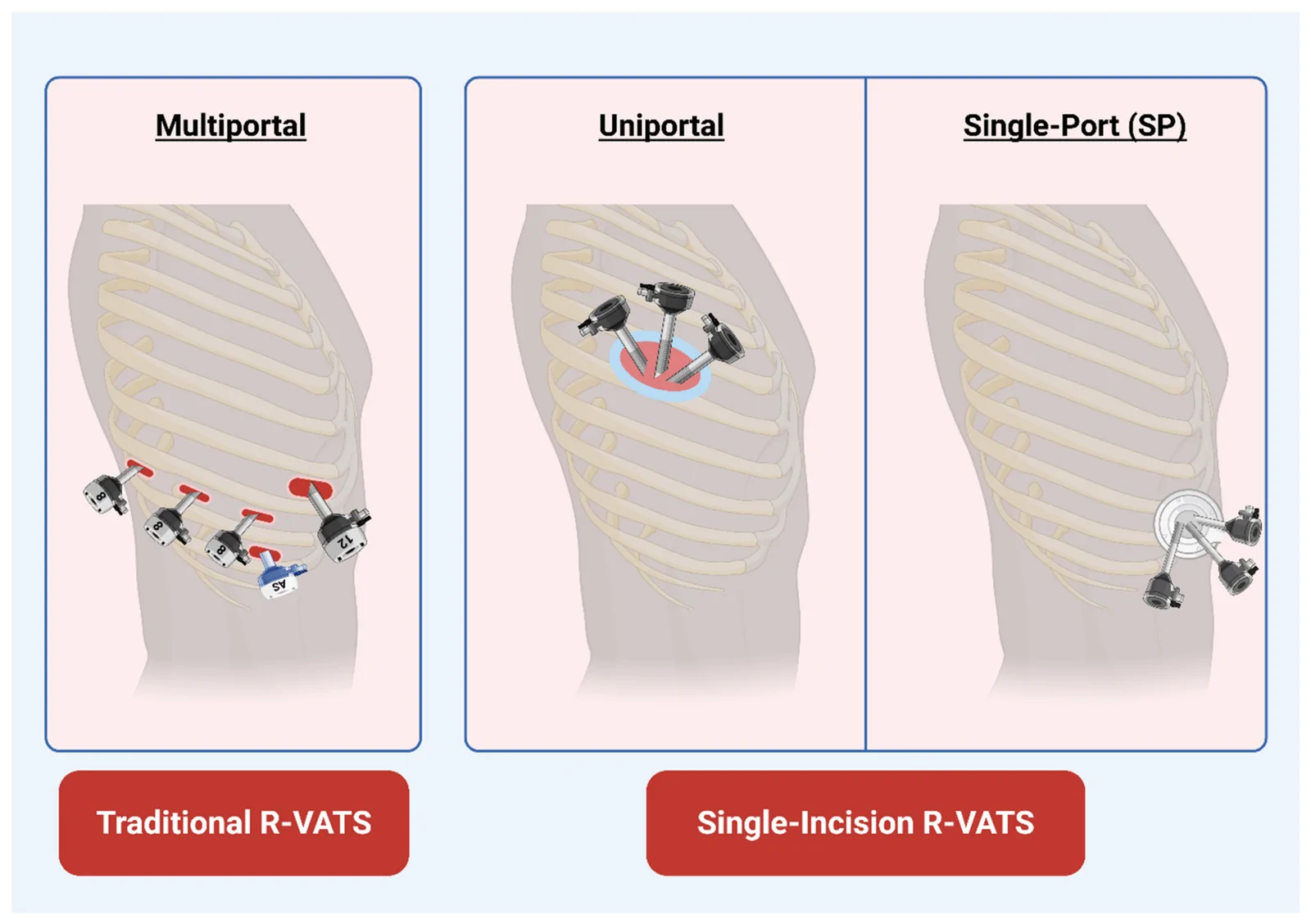
Representative port placements for traditional multiportal R-VATS and single-incision R-VATS. Multiportal R-VATS for lobectomy typically requires four ports (three 8 mm trocars positioned along the 8th intercostal space and one 12 mm trocar usually one rib space above) as well as an AirSeal port (typically one–two rib spaces below). Uniportal trocars are advanced through a single 3–5 cm incision in the fourth or fifth intercostal space. Single-port (SP) R-VATS uses the da Vinci SP platform, docked via a subcostal port.

The robotic platform has accelerated the resurgence of the uniportal approach (Figure 2). In uniportal R-VATS (uR-VATS), the robotic platform is docked through a multi-station port via a single incision [21]. Two distinct technical approaches are utilized for single-incision R-VATS. One approach is the pure uniportal (uR-VATS), which employs the da Vinci Xi system docked through a single intercostal incision, while the other uses the da Vinci SP (Single-Port) system with a subcostal 2.5 cm cannula [29,30].

Proponents of uR-VATS highlight several advantages, including lower perioperative analgesia requirements and LOS, as well as a potentially improved safety profile, including the ability to rapidly undock and emergently convert to a VATS or open approach [31,32]. Compared with uniportal VATS, uR-VATS has equivalent safety and oncologic outcomes [33]. In its nascency, uR-VATS has been shown to be safe for standard anatomic resections and has even been utilized for larger tumors (>5 cm) with reasonable safety and feasibility [34]. Comparisons between uR-VATS and mR-VATS are under active investigation. A multicenter European study of 101 uR-VATS vs. 101 mR-VATS cases demonstrated equivalent short-term oncologic outcomes, including margin status, lymph node yield, and mortality, with uR-VATS. uR-VATS was also associated with lower complication rates [30]. Importantly, operative time (136 vs. 150 min; *p* = 0.0001) and LOS (four vs. five days; *p* < 0.0001) were slightly shorter in the uR-VATS cohort [30]. These data were further supported by a recent propensity-matched analysis of 339 patients undergoing either subcostal uR-VATS or mR-VATS. In this study, the uR-VATS cohort had significantly shorter operative and console times, as well as lower pain scores during the first three postoperative days [35]. Continued investigation is necessary to characterize the learning curve for uR-VATS, compare setup and maintenance costs, and to evaluate differences in long-term outcomes compared to traditional mR-VATS.

## 5. Guiding the Knife: Emerging Role of Radiomics and Artificial Intelligence in Minimally Invasive Surgical Planning for Early-Stage NSCLC

Artificial intelligence (AI) and radiomics are redefining surgical planning for early-stage NSCLC by layering robust, quantitative data onto traditional one-dimensional, size-based readings of preoperative imaging. These tools have shown potential to extract imaging phenotypes associated with histology, staging, prognosis, and treatment response, enabling improved preoperative planning and prognostication for precision surgery [36,37]. Nakamura et al. recently investigated 324 patients who underwent resection for small adenocarcinomas and found that AI assessment improved tumor size measurement and identified pathologic non-invasive adenocarcinoma with a specificity of 98.3%, supporting more confident selection of patients for parenchymal-sparing resection [38]. Furthermore, AI assessment of ground-glass nodules was superlative, with a positive predictive value of 98.5% [38]. Similarly, radiomics may also address features that impact the extent of resection. Zhang et al. developed a novel CT-based radiomics model to predict high-grade patterns that are typically associated with more aggressive disease. In comparison to either a radiomics-only or clinical-only model, a combined model performed best at predicting high-grade patterns on CT for both sub-2 cm lesions and mixed GGNs, consistent with multiple other studies [39,40,41,42,43]. Major high-risk features on CT identified by these models included larger tumor size, spiculation, solid component, pleural indentation, lobulation, vacuolar signs, and presence of an air bronchogram [40,41,42,43]. Overall, these investigations into the implementation of AI-based radiomic models suggest that they may be superior to clinical assessment alone in identifying patients who may be better candidates for sublobar resection vs. formal lobectomy, and patients who may potentially benefit from neoadjuvant or adjuvant therapy based on imaging-based risk profiles.

AI/radiomics has also enhanced the assessment of occult nodal metastasis. Underestimation of occult lymph node metastasis is reported in up to 7% of patients with subcentimeter Stage 1A tumors [44] and up to 20.2% of tumors up to 5 cm in size [45]. In a multicenter study of 752 patients with cN0 early-stage NSCLC, Zhao et al. developed a machine-learning model with a five-tier normal metastasis risk stratification scheme ranging from 3.7% for very-low-risk and up to 40% for very-high-risk cohorts with AUC of >0.80. Their model identified consolidation-to-tumor ratio, specific radiomics-guided features, and lobulation as critical predictors of occult nodal disease [46]. Li et al. demonstrated through a comprehensive meta-analysis that CT and PET/CT-based radiomics predicted lymph node metastasis with AUC of 0.90 [47]. Implementation of such models prior to minimally invasive resection may be instrumental in guiding which patients with clinically node-negative tumors may benefit from aggressive (potentially with more surgical risk) lymphadenectomy vs. more conservative sampling. Clinical trials and further prospective investigations into the long-term oncologic outcomes comparing traditional imaging analysis vs. AI/radiomics model-enhanced analyses are necessary.

AI and radiomics implementation have been particularly promising for segmentectomy planning. In these cases, the major concern is no longer whether a lesion is resectable, but whether it can be treated with limited resection of involved bronchopulmonary segments while achieving the same oncologic outcomes as a lobectomy. It is important to note that despite the prior landmark trials showing non-inferiority of sublobar resection, a recent post hoc analysis of the JCOG0802 trial has raised the question of whether the risk of local relapse after segmentectomy is significantly higher than after lobectomy [48]. In a large cohort of patients with Stage IA NSCLC, Na et al. demonstrated that a CT-based deep learning model, which incorporated 80 CT features as well as clinical and radiologic data, predicted 2-year freedom from recurrence post-segmentectomy with a sensitivity of 87.4%, substantially outperforming the Japan Clinical Oncology Group (JCOG) criteria alone, which had a sensitivity of 37.6% [49]. Beyond oncologic selection, AI is also improving technical aspects of oncologic surgery: both Chen et al. and Lee et al. showed improvements in anatomic identification, reduced surgeon error, and reduced surgeon workload when AI-driven reconstruction or virtual navigation software was used for surgical planning [50,51].

Despite the promise of AI and radiomics, several important limitations must be considered. First, much of the literature discussed reflects lower-level evidence and comprises primarily retrospective, single-institution studies with highly selected cohorts. Even among the high-performing models discussed, substantial variability in CT acquisition parameters, segmentation strategies, and extraction pipelines exists; these make external generalizability and reproducibility challenging. Furthermore, practical barriers, such as time and expertise for image curation, tumor segmentation, and costs for healthcare systems, must be considered.

Overall, AI and radiomics have shown utility in augmenting rather than replacing oncologic and surgical decision-making, allowing physicians to match the extent and execution of resection in NSCLC. While further investigations into the long-term oncologic benefits and feasibility of AI/radiomics-enhanced models are necessary, these studies suggest the potential for AI/radiomic models to augment clinicians’ assessments rather than supplant them.

## 6. The Price of Precision: Understanding the Costs of Robotic Thoracic Surgery and the Challenges for Widespread Implementation

As the utility, clinical indications, and technical advances for R-VATS continue to evolve, the crucial question of its feasibility for widespread adoption remains. Disadvantages of R-VATS include a steep initial learning curve of the platform, lack of haptic feedback while handling fragile tissue, and generally longer operative times [52,53]. Furthermore, in the earlier years of R-VATS, bleeding and cardiovascular complications were noted to be more frequent compared to traditional VATS [54]. It is important to note that as familiarity and training with the robotic platform continued to increase in recent years, the bleeding risk with R-VATS was comparable to, if not lower than, that of VATS [55]. A major barrier to the widespread adoption of R-VATS is cost. Regardless of specialty, the robotic platform is itself extremely expensive [56,57]. In addition, the costs and resources required to develop a functional, efficient surgical robotic practice are substantial. Furthermore, recruiting and training robotic-certified scrub technicians, nurses, and bedside assistants, as well as annual servicing and maintenance costs, markedly increase the overhead cost of setting up a robotic thoracic practice [58]. Even at an individual case level, each robotic instrument has a limited lifespan and can cost up to 2000 dollars per case. A micro-costing analysis from Ireland found that R-VATS cost an average 1754 EUR (≈$2000) more than VATS and 739 EUR (≈$850) more than open thoracotomy, driven largely by consumable and staffing costs [59]. Furthermore, Park et al. conducted a propensity-matched analysis of 268 patients at a single institution and demonstrated that despite the numerous clinical advantages previously discussed, R-VATS was independently associated with higher overall cost [60]. Overall, numerous such analyses have demonstrated that R-VATS may be significantly more costly than conventional VATS or open approaches [57,60,61].

How do medical practices reconcile the advantages of the robotic platform discussed earlier with these significantly higher costs? A systematic review found that while R-VATS costs an average of $3000–4000 more per case (due to robotic instruments and longer operative time), high volume centers (>300 cases) compensate for these higher costs through shorter hospital stays, reduced rates of readmission, and fewer complications compared to non-robotic cases [62]. Furthermore, of all robotic cases at a single institution, Abbas et al. found that thoracic operations had the highest net margin [63]. Similarly, Harrison et al. prospectively analyzed 365 R-VATS cases and found that costs decreased by £640 per case and became comparable to VATS as surgeons progressed through the learning curve, largely driven by reductions in operative time [64].

Collectively, these investigations highlight the complexity of evaluating the economic value of R-VATS, as upfront procedural costs need to be weighed against potential downstream benefits, including reduced complications, shorter hospital stays, fewer readmissions, and increased hospital backfill from other surgical cases. Indeed, Chen et al. showed that R-VATS was cost-effective for the average payor in the Chinese healthcare system relative to open surgery over a 5-year horizon in terms of quality-adjusted life-years, corresponding to an incremental cost-effectiveness ratio (ICER) of $10,967.41 per quality-adjusted life-year [65]. While these findings provide important insights, current studies comparing the cost-effectiveness of R-VATS and VATS are mainly based on retrospective analyses and decision-analytic models, which reflect Level 4 or 5 evidence, at best. The RAVAL trial is expected to address this gap in research by providing the first prospective, randomized economic analysis comparison of R-VATS and VATS, while incorporating cost-utility data alongside clinical outcomes [66].

## 7. Future Direction

The priority of most future investigations into each of the advancements discussed herein is firmly rooted in enhancing the safety and ease-of-access of the R-VATS platform. Single-anesthetic marking protocols, uniportal approaches, and complex analyses in the AI/radiomics space all started at high-volume centers with uniquely experienced surgeons and clinicians who had the foundation on which to innovate. Thus, the future of minimally invasive pulmonary surgery begins with universal robotic training during surgical residency training. This must be further expanded upon during cardiothoracic fellowship.

Robotic training and exposure are highly variable among general surgery residencies, but are increasingly incorporated into the training paradigm with an estimated 70% of programs offering a dedicated robotics curriculum [67]. While safe robotic surgical principles are universal between various subspecialties, thoracic robotic surgery is particularly challenging for several reasons. One major factor is the paucity of working space in the chest, despite insufflation. The thoracic cavity simply does not expand to the same degree as the abdomen with insufflation. This is often the first aspect of robotic thoracic surgery that trainees need to adjust to coming from general surgical training. Furthermore, the chest cavity contains inherently high-risk organs (i.e., pulmonary vasculature, nerves, the trachea, the esophagus, the heart, and great vessels) that are at risk of iatrogenic injury with potentially fatal consequences. As such, guided mentorship and graduated autonomy are of paramount importance to training an aspiring thoracic surgeon to complete a robotic pulmonary resection safely and efficiently. The Society of Thoracic Surgeons Task Force on Robotic Thoracic Surgery recently published the consensus recommendations for a standardized curriculum to ensure that the next generation of cardiothoracic surgeons are ready to utilize R-VATS safely and efficiently. These guidelines include encouragement of dual-console systems for real-time training; advocation for a trained bedside assistant while trainees are at the console; implementation of virtual and wet-lab simulations; development of standardized evaluation criteria; and establishment of key milestones for major aspects of a pulmonary resection [68]. It is only after firmly establishing a baseline level of comfort and experience with the robotic platform that the thoracic oncology community can build upon the unique opportunities to streamline and enhance patient care on a broader scale.

## 8. Conclusions

The future of minimally invasive thoracic surgery is evolving rapidly. The advent of robotic pulmonary resection represents a paradigmatic shift in how lung cancer can be treated with excellent oncologic outcomes, while minimizing postoperative morbidity. As more patients may be amenable to less aggressive sublobar resections, and others may become surgical candidates due to advancements in immunotherapy, robotic pulmonary resection remains well-positioned as the preferred approach for most patients with resectable NSCLC. Single-anesthetic marking and simultaneous resection of concerning pulmonary nodules may accelerate the rate at which the thoracic oncology community can diagnose and treat early-stage disease. AI/radiomics-enhanced analyses of preoperative imaging may identify those patients with higher-risk disease biology who may benefit from perioperative systemic therapies. Furthermore, advancements in uniportal robotic resection may further improve patient outcomes and minimize postoperative pain and cosmetic burden. At the same time, as the adoption of robotic pulmonary resection continues to expand, we must remain cognizant of the logistical and financial costs of implementing robotic thoracic programs across healthcare systems. As ongoing investigations suggest that operating room efficiency (directly predicated on expertise and experience with robotic surgery) may significantly defray costs, we must continue to emphasize training the next generation of cardiothoracic surgeons to perform robotic pulmonary resections safely and efficiently.

## Data Availability

No new data were created or analyzed in this study. Data sharing is not applicable to this article.
